# Effect of CMT and MIG Welding on Microstructure and Mechanical Properties of Al-Zn-Mg-Cu-Er-Zr Alloy

**DOI:** 10.3390/ma18204688

**Published:** 2025-10-13

**Authors:** Wu Wei, Yijie Sun, Chao Zhang, Limin Zhai, Peng Wang, Li Cui, Shengping Wen, Wei Shi, Xiaorong Zhou

**Affiliations:** 1 State Key Laboratory of Materials Low-Carbon Recycling, Beijing University of Technology, Beijing 100124, China; 2 Institute of Corrosion Science and Technology, Guangzhou 510530, China; 3 Department of Materials, The University of Manchester, Manchester M13 9PL, UK

**Keywords:** Al-Zn-Mg-Cu-Er-Zr alloy, metal inert gas arc welding, cold metal transition welding, microstructure, mechanical properties

## Abstract

Cold metal transfer (CMT) welding and metal inert gas (MIG) arc welding of a novel Al-Zn-Mg-Cu-Er-Zr alloy are systematically analyzed. The effect of the two welding processes on the morphology, microstructure, and mechanical properties of welded joints was investigated. The evolution of the microstructures and grain structures in the welded joints is studied using an optical microscope (OM), X-ray diffraction (XRD), and scanning electron microscopy (SEM) with energy dispersive spectroscopy (EDS). The results show that both welding methods obtain well-formed full-penetration welds, and the width of the heat-affected zone (HAZ) of CMT welding is smaller than that of MIG welding. The two welded joints reveal coarse cellular grain structures with precipitates of η (MgZn_2_), Al_3_Er, and S (Al_6_CuMg_4_) secondary phases. The average grain size of the weld metal in the cold metal transfer welding (12.96 μm) joint is much finer than that of the metal inert gas arc welding joint (22.63 μm), with a higher proportion of high-angle grain boundaries (HAGBs). The hardness of cold metal transfer welding and metal inert gas arc welding weld zones is 103.9 HV and 92.6 HV, respectively, and the tensile strength of the joint is 334.0 MPa and 270.3 MPa, respectively.

## 1. Introduction

The novel Al-Zn-Mg-Cu-Er-Zr alloy is an ultra-high strength corrosion-resistant aluminum alloy [1,2,3]. The welding process was optimized to provide a theoretical basis for the engineering application of the new Al-Zn-Mg-Cu-Er-Zr alloy in the far-reaching sea field. It should be noted that the addition of Er and Zr elements significantly improves the corrosion resistance of the alloy through microstructure refinement and the formation of stable precipitates, making it suitable for marine applications [4,5,6]. The connection of deep-sea pressure-resistant pipe with a large thick wall and its structural parts inevitably leads to welding requirements.

At present, some studies exist on the welding of conventional high-strength Al-Zn-Mg-Cu alloys. Yeni et al. [7,8] investigated the microstructure and mechanical properties of metal inert gas arc (MIG) and tungsten inert gas (TIG) joints in a 6 mm thick 7075-T6 alloy. The results showed that the weld and heat-affected zone (HAZ) grains were significantly coarsened compared with the base material (BM). The hardness value decreased significantly, and the strength of MIG joints only reached 43% of the BM. Liu et al. [9] compared laser welding with TIG welding for 7075-T6 aluminum alloy and found that laser-welded joints exhibited higher tensile strength and hardness than TIG-welded joints, However, significant joint softening still occurred in both welding methods compared to the base material, indicating that the fundamental challenge of weld zone softening in high-strength aluminum alloys remained unresolved. Elrefaey [10] applied cold metal transfer (CMT) welding and MIG to weld 7075 aluminum alloy, and the results showed that both welding methods obtained good joints, but CMT joints had better tensile strength and elongation. However, studies on welding the novel Al-Zn-Mg-Cu-Zr-Er alloys are scarce. Zhang et al. [11] conducted a comparative study on Al-Zn-Mg-Cu-Zr-Er alloys’ TIG welding and laser welding. They found that due to the vaporization of Zn and Mg during welding and the precipitation of the T-phase in the weld, the solid saturation of the matrix decreased, the precipitation-strengthening effect of the base material disappeared, and the hardness of the weld was significantly lower than that of the BM and HAZ. This indicates that compared to the conventional high-strength Al-Zn-Mg-Cu alloy, the new Al-Zn-Mg-Cu-Zr-Er-alloy-welded joint has more serious softening, which limits its further application in the engineering field. Therefore, investigating the softening behavior of the novel Al-Zn-Mg-Cu-Zr-Er alloy joint is of great application value. MIG welding remains one of the most widely used, mature, and cost-effective industrial processes. However, issues such as high heat input and severe workpiece deformation limit its application in precision welding of thin-walled components [12]. CMT welding is a novel welding process developed based on droplet short-circuit transition. Compared with MIG welding, CMT welding has apparent advantages such as precise adjustment of arc length, low heat input, no splash transition, and high welding bridging ability, which is conducive to restraining the coursing of weld grains and the burning loss of alloying elements [13,14]. It is applied to welding new Al-Zn-Mg-Cu-Er-Zr alloy to improve the degree of joint softening. However, the research reports on CMT welding of new Al-Zn-Mg-Cu-Er-Zr alloy are limited, particularly regarding joint-softening behavior, which warrants further exploration.

Therefore, using the novel Al-Zn-Mg-Cu-Er-Zr alloy plate butt joint as the research object, a comparative study on the forming, microstructure, and mechanical properties of CMT and MIG welding joints was carried out. The welding process was optimized to provide a theoretical basis for the engineering application of the new Al-Zn-Mg-Cu-Er-Zr alloy in the far-reaching sea field.

## 2. Materials and Methods

The base material is a new 3 mm thick T6 Al-Zn-Mg-Cu-Er-Zr alloy plate, and AlMg_6_Zr welding wire with a diameter of 1.6 mm is selected, and its chemical composition are shown in Table 1. Before welding, the grease and oxide film on the specimen surface were removed by mechanical grinding and chemical methods. The joint adopts a plate-docking mode with a gap of 1 mm and is protected by 99.99% argon gas with a gas flow rate of 18 L/min. The welding process parameters were optimized to obtain complete penetration welds: in CMT welding, the current was 85 A, the arc voltage was 12.8 V, and the welding speed was 6 mm/s. During MIG welding, the current is 120 A, the arc voltage is 18.8 V, and the welding speed is 9 mm/s.

The welded joint was processed by wire cutting. After grinding and polishing, the joint was etched with Keller reagent to prepare the metallographic sample. LEXT OLS4100 laser scanning confocal microscope (Olympus, Tokyo, Japan)was used to observe the metallographic structure, Gemini SEM 300 scanning electron microscope (Carl Zeiss, Oberkochen, Germany) and EDS were used to analyze the welded joints and fractures, and D8 Advance was used to analyze the phase of the joints. HVS-1000 microhardness tester (LECO, St. Joseph, MI, USA) was used to test the microhardness values in different joint areas. The tensile specimens were extracted from the welded joints by wire electrical discharge machining, with the gauge section oriented perpendicular to the welding direction. The specimens were carefully positioned to ensure the weld centerline was located at the middle of the gauge length, with the heat-affected zone (HAZ) symmetrically distributed on both sides. The loading load was 100 gf, and the loading time was 15 s. Tensile test at room temperature was carried out with the MTS810 servo material machine (MTS Systems Corporation, Eden Prairie, MN, USA), and tensile specimens were prepared according to the GB/T 2651-2008 welding [15] joint tensile test method. The transverse tensile test of the joint was carried out at the loading rate of 1 mm/min, and the average values of the tensile strength and elongation of the three groups of samples were taken.

## 3. Results and Discussion

### 3.1. Weld Surface Forming and Cross-Section Appearance

**Figure 1:**
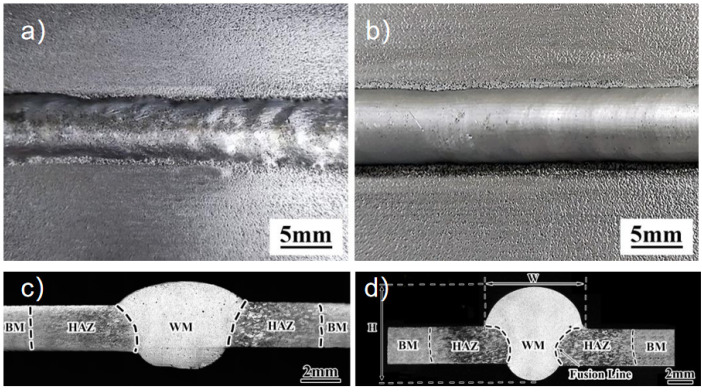
(**a**) Weld appearance of MIG welding; (**b**) weld appearance of CMT welding; (**c**) cross-section of MIG welding; and (**d**) cross-section of CMT welding.

Figure 1 shows MIG and CMT welds’ surface forming and cross-sectional morphology. It can be seen from Figure 1a,b that the surface of the two welds has a regular fish-scale pattern, uniform weld width, no apparent cracks and other welding defects, and the surface of the weld is well-formed. The cross-sectional morphologies of the two joints are shown in Figure 1c,d. A fully permeable weld was obtained. The MIG weld width is 7.2 mm, the depth is 4.0 mm, and the weld-forming coefficient is 1.8. The welding width of the CMT weld is 8.5 mm, the melting depth is 4.5 mm, and the forming coefficient of the weld is 1.9. Therefore, the weld form coefficient of CMT- and MIG-welded Al-Zn-Mg-Cu-Er-Zr alloy is similar. Still, the HAZ width of the CMT joint is significantly smaller than that of MIG welding because the heat input of CMT welding is lower than that of MIG welding. In addition, a small number of pores were observed in MIG welds, while no porosity defects were observed in CMT welds, and CMT obtained better weld and joint formation.

### 3.2. Microstructure and Phase Composition of Joint

**Figure 2:**
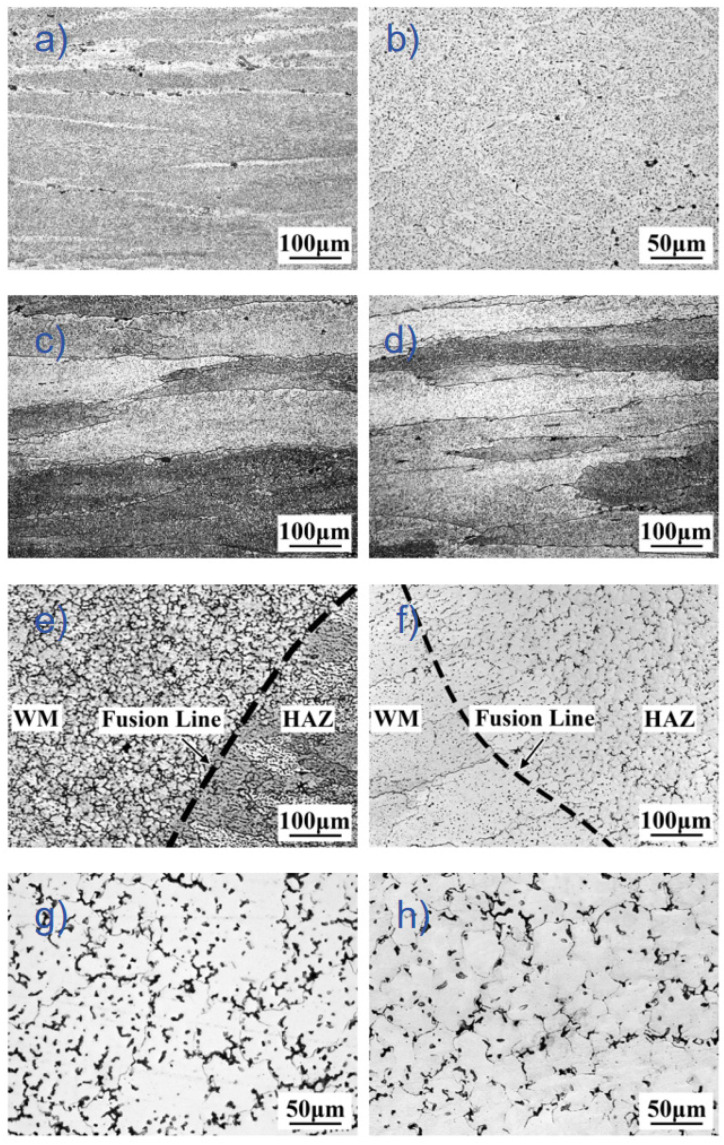
Microstructure of welded joints in Al-Zn-Mg-Cu-Er-Zr alloys. (**a**) BM; (**b**) high-magnification view of BM; (**c**) HAZ of MIG; (**d**) HAZ of CMT; (**e**) FL of MIG; (**f**) FL of CMT; (**g**) WM of MIG; and (**h**) WM of CMT.

The microstructure of the Al-Zn-Mg-Cu-Er-Zr alloy base metal and the joint prepared by the two welding methods are shown in Figure 2. The base material is a typical rolled fibrous structure, as shown in Figure 2a,b. Figure 2c shows the HAZ structure of the MIG joint. The HAZ grain is partially recrystallized due to heat, and the somewhat flat grain’s equiaxed structure of more than ten microns is formed. The HAZ structure of the CMT joint is shown in Figure 2d, with finer grain and almost no recrystallization. Figure 2e,f shows the structures near the fusion zone width, which were determined by measuring the distance from the fusion line to the point where the characteristic weld solidification structure (dendritic or cellular morphology) transitions to the heat-affected zone microstructure. Measurements were taken at multiple locations along the fusion boundary, and average values of 400 μm for MIG and 200 μm for CMT welding were reported. Microstructural analysis of the welds revealed a region of refined grains adjacent to the fusion line in the MIG joint, characteristic of an equiaxed zone (EQZ) commonly observed in Al-Zn-Mg-Cu alloys containing Zr. Based on the examination of the weld cross-sections, the grain size in this region was estimated to be in the range of 5–10 μm t, the so-called EQZ. Although not perfectly equiaxed, this region exhibits significantly more isotropic grain growth compared to the typical columnar dendritic structure in weld fusion zones. EQZ has been found in the welding of some Zr-containing aluminum alloys. EQZ is a unique phenomenon in the weld microstructure of aluminum alloys. Different from the general microstructure of the weld fusion zone, there is no apparent co-crystallization between EQZ and the base metal [16]. The presence of EQZ reduces the amount of the second phase. It leads to a decrease in the joint strength, caused by the severe segregation of the strengthened elements Zn, Mg, and Cu and the rapid cooling and solidification, which makes the remaining solute atoms too late to re-precipitate. The microstructure of the CMT weld is nucleated by the semi-melted base metal grains near the fusion zone and grows towards the weld center in the direction of the fastest heat dissipation; that is, the standard joint crystallization occurs. Figure 2g shows the microstructure of the MIG weld. The dendrite morphology is similar to CMT welds, but the dendrite fracture particles are more numerous and densely distributed. Figure 2h shows the metal structure of the CMT weld. The primary dendrite fracture is distributed uniformly in the α-Al matrix in granular form.

**Figure 3:**
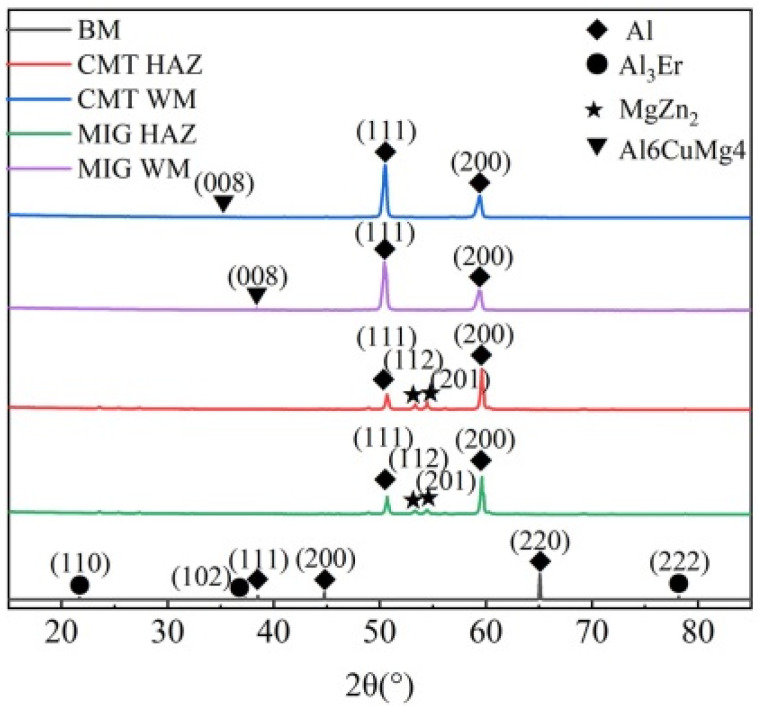
XRD patterns of different regions of Al-Zn-Mg-Cu-Er-Zr alloy joints.

Figure 3 shows the XRD pattern of the base material, HAZ and weld area of MIG and CMT welded joints. It can be seen that the base material Al-Zn-Mg-Cu-Er-Zr alloy is composed of the Al phase, η (MgZn_2_) phase, and Al_3_Er phase, among which η phase is the main strengthening phase, which has a significant effect on the aging strengthening of the alloy, and Al_3_Er phase is a stable L12 structure, which has a high melting point and good stability. It can be used as the core of heterogeneous nucleation, improving the nucleation rate and refining grains [17]. The HAZ structure of CMT and MIG joints formed the Al and η phases. Still, the weld zone was composed of an Al phase and a small amount of Al_6_CuMg_4_, and no η phase was found, which was due to the extensive heat input of MIG welding, resulting in a large amount of Mg and Zn elements in the weld, which significantly reduced their content and was not enough to form the η phase.

### 3.3. Grain Morphology and Grain Orientation Distribution of Weld Microstructure

**Figure 4:**
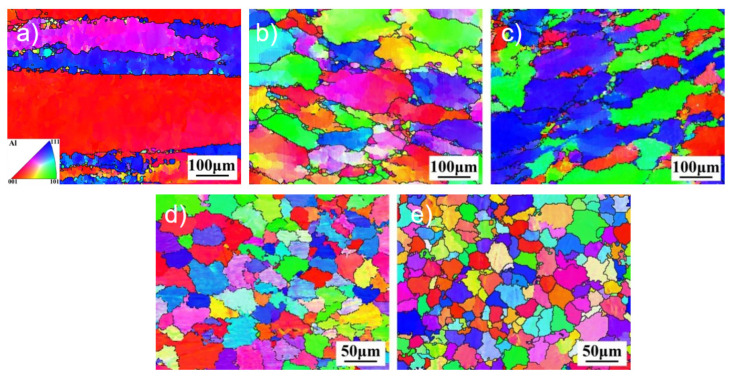
Grain orientation map of Al-Zn-Mg-Cu-Er-Zr alloy joints in different regions. (**a**) IPF of BM; (**b**) IPF of MIG HAZ; (**c**) IPF of CMT HAZ; (**d**) IPF of MIG WM; (**e**) IPF of CMT WM.

To further analyze the grain morphology and size of the joint structure, the HAZ MIG and CMT welds of Al-Zn-Mg-Cu-Er-Zr alloy base metal, MIG and CMT joints were tested by Electron Backscatter Diffraction(EBSD) technology, and the grain size and grain orientation of the weld structures were quantitatively analyzed. The EBSD microstructure results are shown in Figure 4. As can be seen from Figure 4a, the grain of the base material structure is significantly elongated along the rolling direction, which is a typical rolling structure with a preferred orientation. Careful observation also found refined equiaxed grains on the grain boundary. The ratio of the length to the diameter of the HAZ grain of the joint decreased significantly. It changed from the coarse, flat shape of the base material to the fine-strip shape, which the action of welding heat input may cause, and the recovery and recrystallization occurred in HAZ. It can be seen from Figure 4d,e that the microstructure of both welds presents a uniform distribution of equiaxed crystals, but the grain size of CMT welds is smaller.

**Figure 5:**
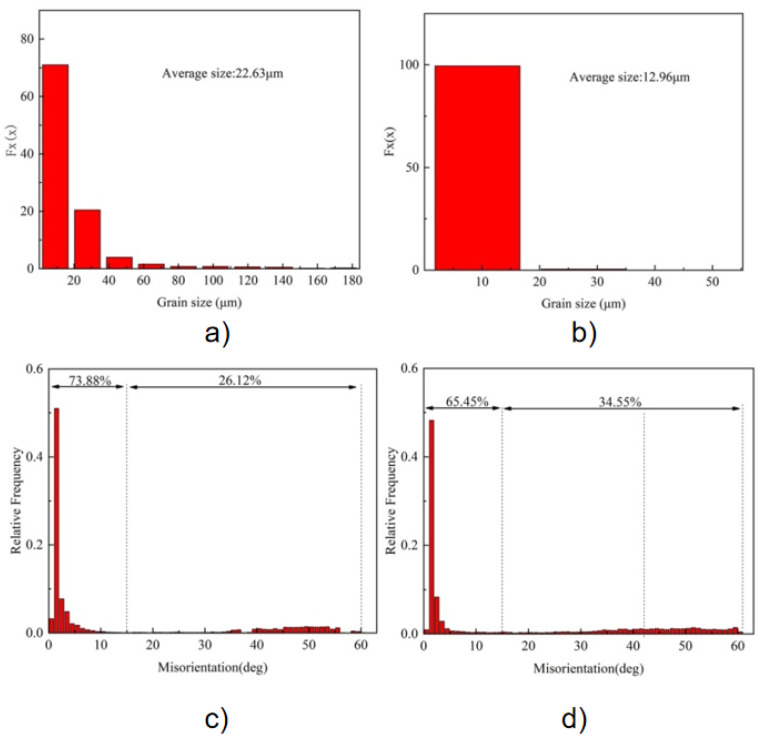
Statistical results of grain size and grain boundary orientation difference in Al-Zn-Mg-Cu-Er-Zr alloy WM. (**a**) Grain size distribution of MIG WM;(**b**) grain size distribution of CMT WM; (**c**) distribution of grain boundary orientation difference in MIG WM; and (**d**) distribution of grain boundary orientation difference in CMT WM.

The orientation of grains is randomly distributed, and there is no apparent preferred orientation. The grain size distribution in Figure 5a,b shows that 71.0% of the MIG welds have grain sizes smaller than 20 μm, with an average grain size of 22.63 μm, while 99.0% of the CMT welds have grain sizes ranging from 2.5 μm to 17.0 μm, with an average grain size of 12.96 μm. Compared with MIG welds, the average grain size of CMT welds is reduced by 42.7%, indicating that CMT welding significantly refines the weld microstructure and has better uniformity. The grain orientation distribution of the two weld structures is shown in Figure 5c,d, where the grain boundary orientation difference between 2° and 15° is defined as the Low-angle grain boundaries (LAGBs). High-angle grain boundaries (HAGBs) are defined as orientation differences more significant than 15°. As can be seen from the figure, the large angle grain boundary ratio of MIG and CMT weld microstructure grains is 26.12% and 34.55%, respectively, indicating that the grains of CMT weld microstructure have more significant angle grain boundaries. Generally speaking, the smaller the grains in the weld, the larger the large-angle grain boundaries, and the more obvious the effect of hindering dislocation movement, the higher the strength [18]. In addition, since dislocation is easy to deposit at grain boundaries, the ratio of large-angle grain boundaries in CMT welds is high. Large-angle grain boundaries can not only change the crack propagation path but also increase the interface energy, thus preventing crack propagation during the fracture process and improving the toughness of joints [19].

### 3.4. The Secondary Phase of the Joint with Different Regional Organization

**Figure 6:**
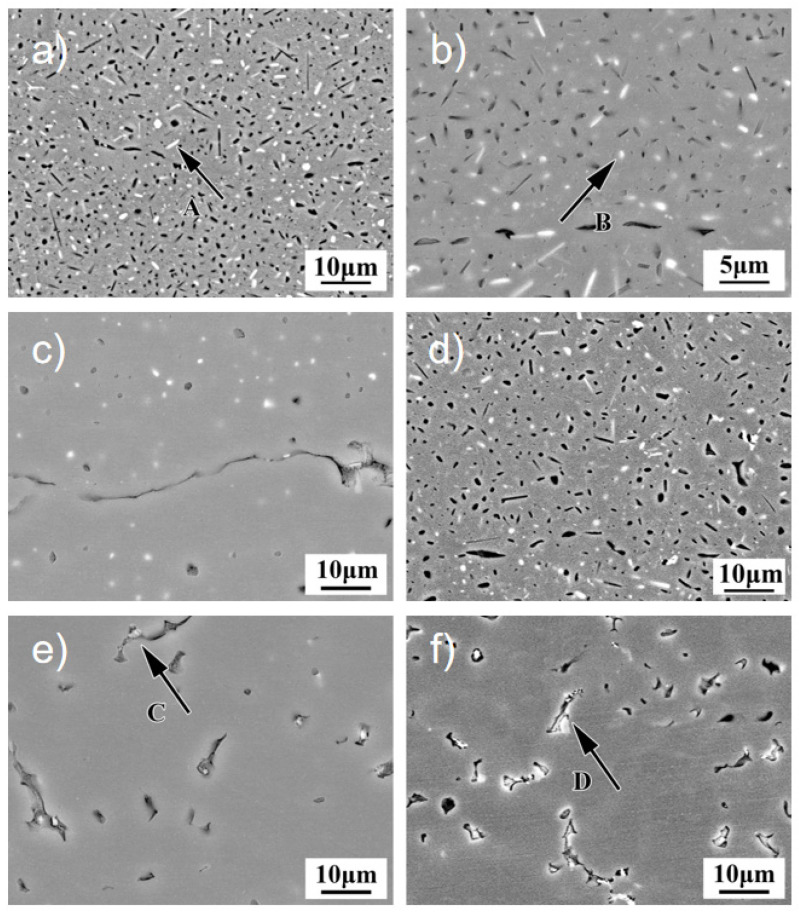
SEM of Al-Zn-Mg-Cu-Er-Zr-alloy-welded joint in different areas. (**a**) BM; (**b**) high-magnification view of BM; (**c**) HAZ of CMT; (**d**) HAZ of MIG; (**e**) WM of CMT; and (**f**) WM of CMT.

Figure 6 shows SEM-backscattered electron images of Al-Zn-Mg-Cu-Er-Zr alloy joints in different CMT and MIG welding regions. Figure 6a,b shows the secondary phase morphology of the base metal region. As can be seen from the figure, the base material structure presents two second phases, namely, the white rod-like phase with a size of about 5 μm and the white granular phase with a size of 1 μm~2 μm dispersed in the matrix. EDS analysis showed that the rod-like second phase enriched Zn and Mg elements, and the granular second phase enhanced Er. These second phases were preliminarily deduced to be the η phase and primary Al_3_Er phase, respectively, and were consistent with XRD analysis results.

Figure 6c shows the backscattered electron image of the CMT joint HAZ tissue. It can be seen from the figure that the amount of second phase precipitated in the HAZ structure is less than that in the base material, and the coarse rod-like second phase completely disappears, while the black and white granular second phase is dispersed in the HAZ structure from 1 μm to 3 μm. This is due to the remelting of the coarse rod-like second phase under welding heat input and the re-precipitation of granular in the subsequent natural aging process. Figure 6d shows the backscattered electron image of the HAZ tissue of the MIG junction. It can be seen that there are a large number of granular and rod-like second phases distributed in the crystal, and the number of second phases is significantly higher than that of CMT joints. But MIG welding has a higher heat input, and the size of the second phase precipitated is significantly larger than that of the CMT joints.

Figure 6e,f, respectively, shows that the weld structure of CMT and MIG joints is mainly based on α-Al solid solution, and many eutectic structures are distributed in grain and dendritic boundaries. After corrosion, pits appear in the shape of vermicular or granular, and the amount of second phase precipitation in the crystal decreases significantly. However, because the heat input of CMT is more minor, the spacing between dendrites is smaller relative to MIG. Like CMT welds, MIG welds exhibit typical as-cast solidified eutectic structures distributed as dendrites in the α-Al matrix. EDS analysis was performed on positions A to D marked in the figure, and the results are shown in Table 2. The rod-like A phase enriched Mg and Zn, and the contents (atomic fraction, the same below) were 1.82% and 3.91%, respectively. Combined with XRD analysis, it is inferred that the A phase may be the MgZn_2_ phase. The content of Er element in phase B is 0.08%, which suggests that phase B is Al_3_Er. The Mg content of C and D grain boundaries is 4.01% and 6.46%, respectively, which is significantly higher than that of the base material, indicating that the grain boundaries are enriched with Mg, which is due to the filling of high-magnesium welding wire to supplement the evaporation and burning loss of Mg elements in the weld. Combined with XRD and the literature, it can be seen that the second phase of the thick long strip in the weld zone is the S(Al_6_CuMg_4_) phase. The coarse second phase consumes the main alloying elements, which weakens the solid solubility of the matrix and the subsequent aging precipitation ability. At the same time, the coarse second phase is not coherent with the matrix, and the size is large, so the strengthening effect is weak.

### 3.5. Mechanical Properties of Welded Joints

**Figure 7:**
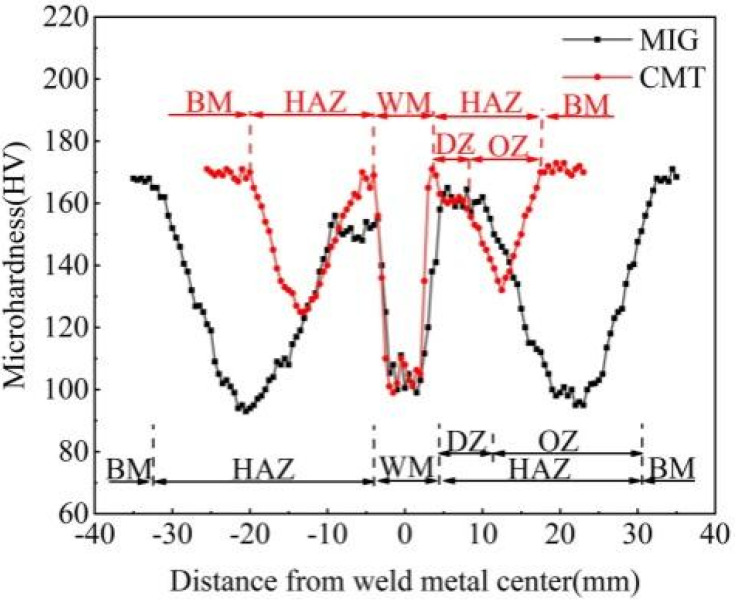
Microhardness profile of joints of Al-Zn-Mg-Cu-Zr alloy produced by CMT and MIG welding.

Figure 7 shows the microhardness distribution in different regions of CMT and MIG joints of Al-Zn-Mg-Cu-Er-Zr alloys. As can be seen from the figure, the hardness values of the two joints are symmetrically distributed along the center of the weld when the base material passes through HAZ and weld. The hardness of the weld and HAZ is significantly reduced compared with that of the base material, and two obvious softening areas appear. The average hardness of Al-Zn-Mg-Cu-Er-Zr alloy base metal is 168.9 HV. After welding, the hardness of CMT weld is 103.9 HV, and the lowest hardness of HAZ is 125.2 HV. The hardness value of MIG weld is 92.6 HV, and the lowest hardness value of HAZ is 97.0 HV. It can be seen that the weld zone and HAZ hardness of the joint are lower than that of the base material, and there is a severe softening phenomenon of the joint. This is caused by the burning loss of Zn and Mg elements in the weld and the re-dissolution of the second phase. In addition, the HAZ hardness of MIG welding is lower than 28.2 HV of CMT welding, and the softening degree is more serious. The CMT weld and HAZ width are minor compared to MIG joints, which reduces the area of joint softening and also helps to improve the degree of joint softening.

Al-Zn-Mg-Cu-Er-Zr alloy CMT and MIG joints have a high hardness zone between the weld and HAZ, that is, the so-called melting zone (DZ), with hardness values of 165.3 HV and 160.1 HV, respectively. This is because most of the initial second phase is dissolved back into the matrix by the action of welding thermal cycling. In the subsequent cooling and natural aging process, the dispersed small second phase will be precipitated, increasing the hardness value of the melting zone. In addition, the HAZ hardness of CMT and MIG joints decreases to 125.2 HV and 97.0 HV at 13.5 mm and 20.5 mm from the weld center, respectively, and an over-age softening zone (OZ) appears. The reason for the over-aging area’s softening is that the thermal cycle’s peak temperature is higher than the initial dissolution temperature of η’ phase and lower than the complete re-dissolution temperature of η’ and η phases. There are reactions of re-dissolution, coarsening, transformation of η’ to η phase, and re-dissolution of η phase. The matrix contains η’ and η phases, and the precipitation phase is more prominent in size and less in quantity. The precipitation strengthening effect is lower than that of the base material [11].

The tensile properties of Al-Zn-Mg-Cu-Er-Zr alloy base metal, CMT, and MIG joints are shown in Table 3. As can be seen from Table 3, the CMT joint yield strength is 207.0 MPa, the tensile strength is 334.0 MPa, elongation after fracture is 11.5%, and the strength coefficient of the welded joint is 0.60. The yield strength of the MIG joint is 155.0 MPa, the tensile strength is 270.3 MPa, the elongation after fracture is 9.0%, and the strength coefficient of the welded joint is 0.47. Therefore, the tensile properties of CMT joints are significantly better than those of MIG joints.

**Figure 8:**
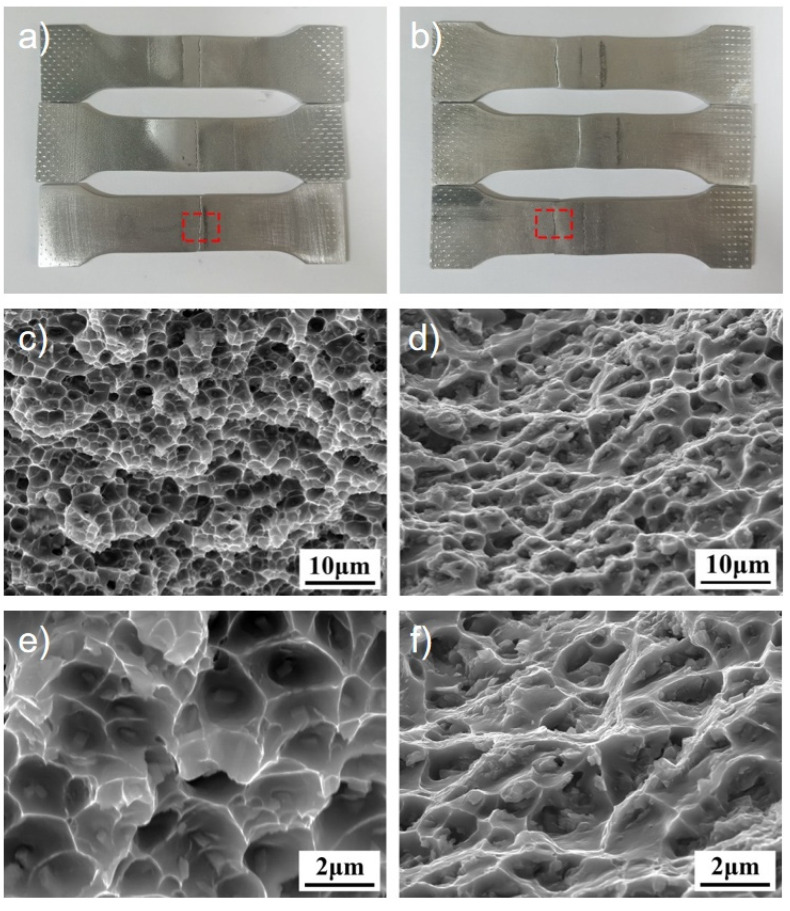
Fracture morphology of welded joints in Al-Zn-Mg-Cu-Er-Zr alloys. (**a**) Macro morphology of tensile fractured sample of CMT joints; (**b**) macro morphology of tensile fracture specimen of MIG joints; (**c**) fracture morphology of CMT welding joints; (**d**) fracture morphology of MIG welded joints; (**e**) high-magnification micro fracture morphology of a CMT welded joint; and (**f**) high-magnification micro fracture morphology of MIG welded joints.

Figure 8 shows the tensile fracture morphology of MIG and CMT joints of Al-Zn-Mg-Cu-Er-Zr alloy. As seen from Figure 8a,b the two joints have no obvious plastic deformation before fracture. The fracture of the CMT joint is in the weld zone, and the rupture of the MIG joint is in the over-aging zone of HAZ, which is consistent with the result of the hardness distribution of the joint. The results show that the weakest area of the CMT joint is the weld area, while the MIG joint’s weakest area is HAZ’s over-aging area. Figure 8c,d correspond to the fracture morphologies within the red boxes in Figure 8a,b. As can be seen from the figure, there are elongated dimples, tearing edges, and broken second phase particles on the fracture surface, showing a mixed fracture mode of toughness and brittleness. The size of the dimple is ten microns, and the dimple is shallow, and there is an irregular second phase with a size of 1 μm in the tearing edge and dimple. In the process of stretching, the aluminum matrix’s slip system is easier to start, the second phase in the weld obstructs the movement of the dislocation, and the accumulation near the second phase increases the elastic strain energy. When the elastic strain energy can overcome the binding force of the second phase with the matrix, a new surface will be formed, resulting in new holes. With the continuous increase in tensile stress, the holes will continue to increase until the joint breaks [20]. As can be seen from Figure 8d, the fracture mode of the MIG joint exhibits predominantly brittle characteristics with a rock-sugar appearance, but also shows evidence of limited ductile deformation at grain boundaries, indicating a semi-ductile fracture mode. The high-magnification SEM image in Figure 8f reveals both cleavage facets characteristic of brittle fracture and localized plastic deformation features. The high-power SEM photos are shown in Figure 8f, in which large-size grains coexist with small-size grains, and the tearing edges of the grain boundaries are obvious.

## 4. Conclusions

(1) The new Al-Zn-Mg-Cu-Er-Zr alloy welded by CMT and MIG can obtain well-formed full-penetration joints with similar weld formation coefficients, but the HAZ width of MIG welding is about two times that of CMT welding.
(2) Crystallization and EQZ occur near the fusion lines of CMT and MIG joints of Al-Zn-Mg-Cu-Er-Zr alloys, respectively. CMT and MIG welds are composed of cellular crystals with grain sizes of 12.96 μm and 22.63 μm, respectively. The grains of CMT are significantly refined, and the grain boundaries of CMT welds are 24.43% higher than those of MIG. The second phase in the two kinds of weld structures comprises the η phase, primary Al_3_Er phase, and S phase, but their contents are different. The thick S phase consumes the main alloying element and reduces the mechanical properties of the joint.
(3) The hardness of the Al-Zn-Mg-Cu-Er-Zr alloy is 168.9 HV, the hardness of CMT and MIG weld zone is 103.9 HV and 92.6 HV, respectively, and the lowest HAZ hardness is 125.2 HV and 97.0 HV, and the hardness of weld and HAZ are lower than that of base metal. However, the HAZ softening of MIG joints is more serious than that of CMT joints.
(4) The tensile strength of the Al-Zn-Mg-Cu-Er-Zr alloy MIG joint is 270.3 MPa, the elongation is 9.0%, and the welding strength coefficient is 0.47. The tensile strength of the CMT joint is 334.0 MPa, the elongation is 11.5%, and the welding strength coefficient is 0.60. Compared with the base material, the tensile strength of the two joints is lower than that of the base material. Still, the CMT joint of Al-Zn-Mg-Cu-Er-Zr alloy has higher tensile properties and improves the softening degree of the joint.
(5) The superior mechanical properties of CMT joints over MIG joints are primarily attributed to their lower heat input and controlled solidification. This yields a finer weld grain structure, a narrower and less softened heat-affected zone, a more uniform distribution of secondary phases, and a higher proportion of strengthening high-angle grain boundaries. Collectively, these factors mitigate joint softening and enhance performance in the Al-Zn-Mg-Cu-Er-Zr alloy.

## Figures and Tables

**Table 1:** Chemical composition of Al-Zn-Mg-Cu-Er-Zr alloys and filler wire (wt.%).

	Si	Fe	Cu	Mn	Mg	Zn	Ti	Cr	Zr	Er	Al
**Alloy**	0.09	0.08	0.60	0.30	2.30	7.30	-	-	0.12	0.11	Bal
**Filler**	0.40	0.40	0.05	0.8–0.9	5.5–6.1	0.20	0.02–0.03	0.03	0.10	-	Bal

**Table 2:** Compositions of second phases in the two weld metal and base metal (at. %).

Location	Mg	Mn	Zn	Cu	Er	Al	Second Phase
**A**	1.82	-	3.91	-	-	94.2	Al, MgZn_2_
**B**	0.62	0.16	3.23	2.2	0.08	93.7	Al, Al_3_Er
**C**	4.01	0.53	3.00	0.38	-	92.08	Al, Al_6_CuMg_4_
**D**	6.46	0.23	1.28	0.56	-	92.08	Al, Al_6_CuMg_4_

**Table 3:** Tensile properties of BM, CMT and MIG welded joints.

	Yield Stress (MPa)	Ultimate Tensile Strength (MPa)	Elongation (%)
**Base material**	537.0	568.7	11.5
**CMT joint**	207.0	334.0	11.3
**MIG joint**	155.0	270.3	9.0

## Data Availability

The original contributions presented in this study are included in the article. Further inquiries can be directed to the corresponding author.
